# Supplementary material for: Kinetochore dynein is sufficient to biorient chromosomes and remodel the outer kinetochore
Source: bioRxiv. 2023 Mar 24:2023.03.23.534015. Preprint. [Version 1] doi: 10.1101/2023.03.23.534015 (PMC10055418; doi:10.1101/2023.03.23.534015)
Supplement: Supplement 1 [file NIHPP2023.03.23.534015v1-supplement-1.pdf]

bioRxiv preprint doi: https://doi.org/10.1101/2023.03.23.534015; this version posted March 24, 2023. The copyright holder for this preprint (which was not certified by peer review) is the author/funder, who has granted bioRxiv a license to display the preprint in perpetuity. It is made available under aCC-BY 4.0 International license.

SUPPLEMENTAL FIGURES & LEGENDS

Figure S1

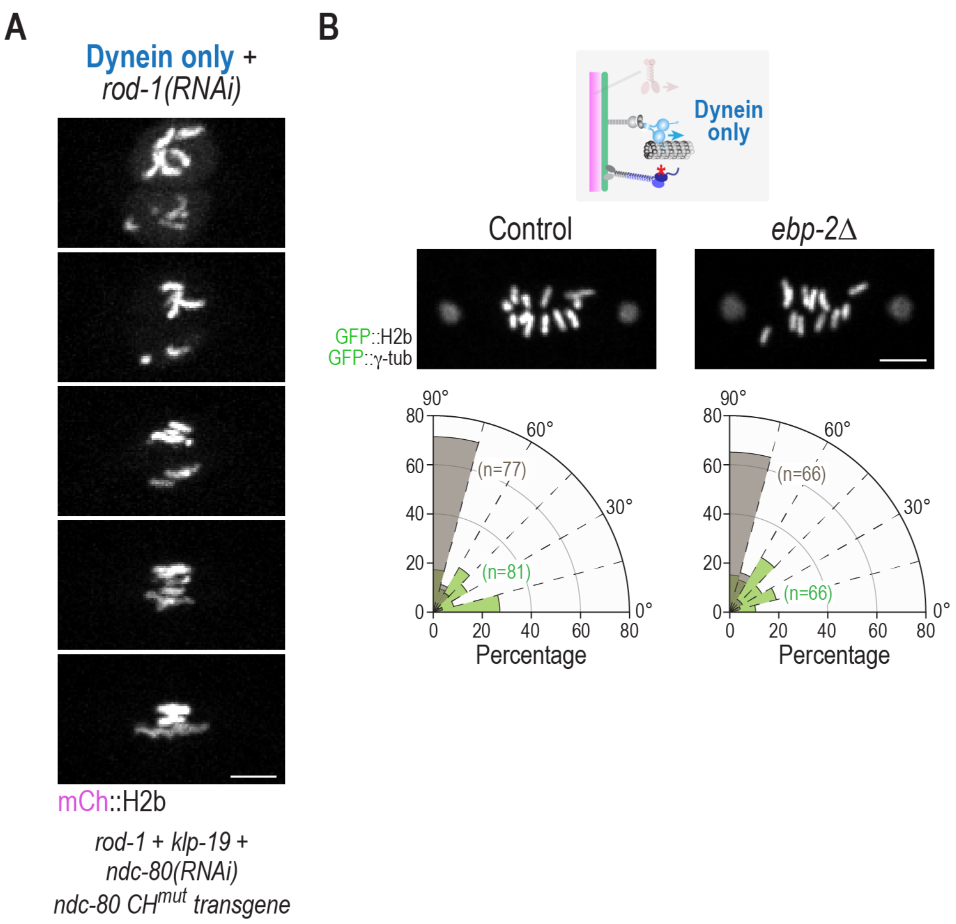

Figure S1. Representative image sequence of ROD-1 depletion in the dynein only state and analysis of *ebp-2*Δ.

(A) Representative images from a timelapse series following removal of ROD-1 in the condition used to generate the dynein only state. Text below the panel indicates the specific perturbations used in this experiment. Quantification of chromosome angle in this condition is shown in *Fig. 2D*. Scale bar, 5 μm.

(B) Analysis of the kinetochore dynein only state in control or *ebp-2*Δ embryos. Loss of EBP-2, which prevent plus end tracking of dynein, has no significant effect on chromosome orientation, indicating that RZZ-recruited dynein is responsible for the orientation function. Scale bar, 5 μm.

bioRxiv preprint doi: https://doi.org/10.1101/2023.03.23.534015; this version posted March 24, 2023. The copyright holder for this preprint (which was not certified by peer review) is the author/funder, who has granted bioRxiv a license to display the preprint in perpetuity. It is made available under aCC-BY 4.0 International license.

Figure S2

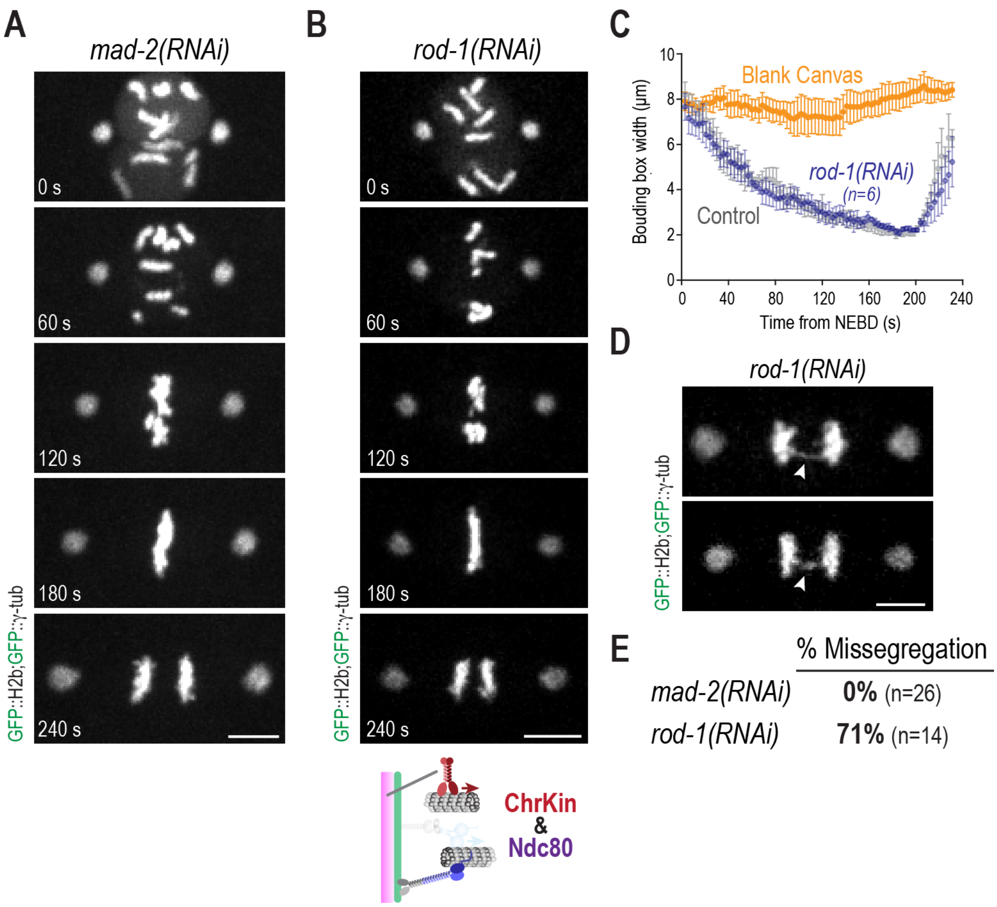

Figure S2. Analysis of checkpoint inhibition and of the chromokinesin–Ndc80 module combination created by removal of kinetochore dynein.

(A) Chromosome dynamics in embryos depleted of the spindle checkpoint protein MAD-2. Loss of MAD-2 does not have any significant effect on chromosome segregation (*see also panel E*). Scale bar, 5 μm. **(B)** Chromosome dynamics in embryos lacking the kinetochore dynein module, where chromokinesin and the Ndc80 module are present. Scale bar, 5 μm. **(C)** Quantification of chromosome dispersion on the spindle performed as in *Fig. 1D*. The Control and Blank Canvas curves are the same as in *Fig. 1D* and are plotted to aid comparison. *n* is number of embryos analyzed. **(D)** Examples of anaphase segregation defects observed in the absence of the kinetochore dynein module. Scale bar, 5 μm. **(E)** Summary of missegregation events observed in anaphase of one-cell embryos. *n* is number of embryos imaged. Inhibition of the spindle checkpoint does not explain the segregation defect observed in the absence of the kinetochore dynein module.

bioRxiv preprint doi: https://doi.org/10.1101/2023.03.23.534015; this version posted March 24, 2023. The copyright holder for this preprint (which was not certified by peer review) is the author/funder, who has granted bioRxiv a license to display the preprint in perpetuity. It is made available under aCC-BY 4.0 International license.

Figure S3

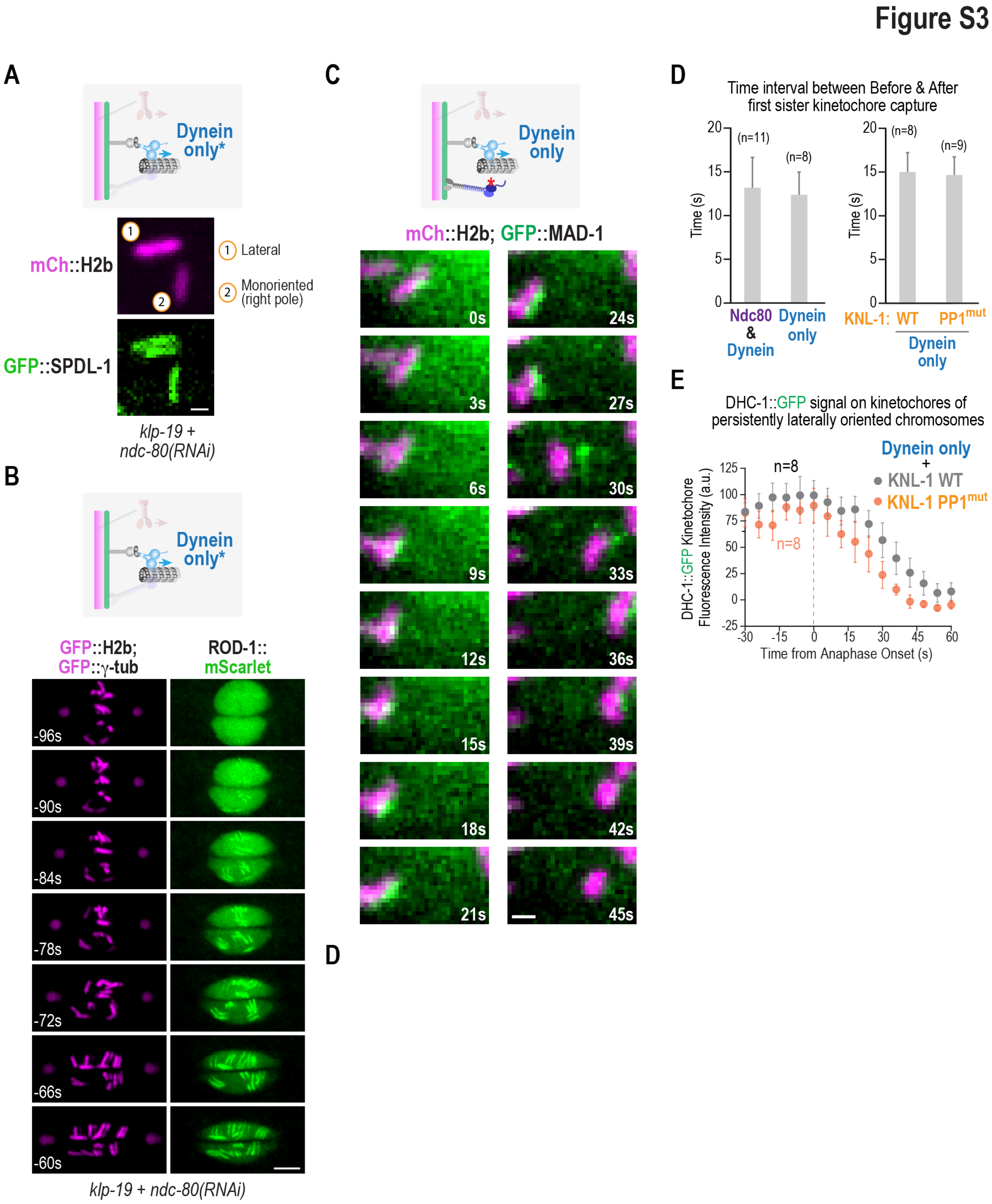

bioRxiv preprint doi: https://doi.org/10.1101/2023.03.23.534015; this version posted March 24, 2023. The copyright holder for this preprint (which was not certified by peer review) is the author/funder, who has granted bioRxiv a license to display the preprint in perpetuity. It is made available under aCC-BY 4.0 International license.

Figure S3. Imaging of GFP::SPDL-1, ROD-1::mScarlet and GFP::MAD-1, and supporting data for analysis of Ndc80 module and protein phosphatase 1 inhibitions.

(A) & (B) Images of *in situ*-tagged GFP::SPDL-1 and ROD-1::mScarlet in *klp-19+ndc-80(RNAi)* embryos. As the mutant NDC-80 transgene was not present, this condition is labeled Dynein Only*. SPDL-1 behaved similarly to DHC-1, in that kinetochore-autonomous removal was observed. However, ROD-1 behaved distinctly–its levels were maintained at oriented kinetochores. Scale bars, 1 μm in *(A)* and 5 μm in *(B)*. **(C)** Image sequence of GFP::MAD-1 in the kinetochore dynein only state. The high signal of GFP::MAD-1 in the spindle region, together with its later recruitment, made imaging its dynamics on single kinetochores challenging. Nonetheless, chromosomes with clear kinetochore MAD-1 signal exhibited orientation-coupled removal from kinetochores. Scale bar, 1 μm. **(D)** Interval between the Before and After timepoints analyzed in *Fig. 5A*. The same interval is also plotted for the condition analyzed in *Fig. 5B*, although DHC-1 signal intensity before orientation was not measured. **(E)** Plot of DHC-1::GFP signal on persistently lateral chromosomes after anaphase onset for the indicated conditions. Error bars are the 95% confidence interval.

bioRxiv preprint doi: https://doi.org/10.1101/2023.03.23.534015; this version posted March 24, 2023. The copyright holder for this preprint (which was not certified by peer review) is the author/funder, who has granted bioRxiv a license to display the preprint in perpetuity. It is made available under aCC-BY 4.0 International license.

METHODS:

C. elegans strains

C. elegans strains are described in *Table S1*. All strains were maintained using standard C. elegans growth media and imaged at 20ºC. Endogenous locus GFP tagging was performed using CRISPR/Cas9[53] at the *dhc-1* locus (dynein heavy chain) and the *klp-19* locus (chromokinesin). For both *dhc-1::gfp* and *gfp::klp-19* the repair template contained two homology arms, a linker sequence (GGRAGSG) and a sequence encoding GFP. GFP integrations were confirmed by PCR. For details on gRNAs, see *Table S2*.

RNA-mediated interference

DNA templates were generated via PCR using the primers as specified in *Table S3*, and subsequently purified using a QIAquick PCR Purification Kit (Qiagen). Single-stranded RNA was generated from each DNA template using a MEGAscript™ T3 and T7 Transcription Kit (Invitrogen), and subsequently purified using a MEGAclear™ Transcription Clean-Up Kit (Invitrogen). Double-stranded RNA (dsRNA) was generated by annealing the single-stranded RNAs at 37°C for 30 minutes[43]. 36-46h before dissection and embryo imaging, the dsRNA was injected into L4 hermaphrodites, which were maintained at 20°C. All RNAi experiments were performed using 1 mg/ml individual dsRNAs, or 1:1 or 1:1:1 mixtures of 1 mg/ml individual dsRNAs.

One-cell embryo fluorescence microscopy and image analysis

One-cell embryos were dissected from adult hermaphrodites in M9 buffer, placed onto a microscope slide containing a 2% agarose pad, and subsequently covered with a 22x22 mm high-precision cover glass (No. 1.5H, Marienfeld).

Embryos were imaged on a spinning-disk confocal (Revolution XD Confocal System; Andor Technology) with a confocal scanner unit (CSU-10, Yokogawa Corporation) attached to an inverted microscope body (TE2000-E, Nikon), illuminated using solid-state 100 mW lasers using either a 60X or 100X 1.4 NA Plan Apochromat oil objective (Nikon), and an EMCCD camera (iXon DV887, Andor Technology) (Desai Lab, San Diego).

One-cell embryos were also imaged on a spinning-disk confocal (CSU-W1 Confocal System, Nikon) with a confocal scanner unit (CSU-W1, Yokogawa Corporation) attached to an inverted microscope body (ECLIPSE Ti2-E, Nikon), illuminated using solid-state 200 mW lasers using either a 60X or 100X 1.4 HP Plan Apochromat oil objective (Nikon), and an sCMOS camera (Prime 95B, Teledyne Photometrics) (Cheerambathur Lab, Edinburgh).

bioRxiv preprint doi: https://doi.org/10.1101/2023.03.23.534015; this version posted March 24, 2023. The copyright holder for this preprint (which was not certified by peer review) is the author/funder, who has granted bioRxiv a license to display the preprint in perpetuity. It is made available under aCC-BY 4.0 International license.

For localization analysis of NDC-80::GFP, GFP::KLP-19 and DHC-1::GFP, 5 x 1.5 µm z-stacks were acquired every 10s and for KNL-1::GFP every 3s. For single chromosome localization analysis of DHC-1::GFP, 5 x 1.5 µm z-stacks were acquired every 3 s starting ~1 min after NEBD and maximum intensity projections (MIPs) generated using Image J (Fiji). Subsequently, the fluorescent background was subtracted and a rectangular box (0.4 x 1.6 µm) fitted adjacent to the mCherry::H2B signal (chromosome) encapsulating kinetochore dynein to obtain the average DHC-1::GFP intensity.

For minimum bounding box (MMB) analysis, 5 x 1.5 µm z-stacks were acquired every 3 s, MIPs generated using Image J (Fiji) and rotated to position the spindle poles horizontally. Fluorescence intensity for all MIPs in the series was normalized, converted to 8-bit and the fluorescence background subtracted. Subsequently, to each MIP image a MMB was fitted (pixel value > 0, i.e. fluorescent signal from GFP::H2B) to measure chromosome positioning.

For chromosome orientation analysis, 5 x 1.5 µm z-stacks were acquired every 2 s for GFP::H2B or 3 s for DHC-1::GFP, MIPs generated using Image J (Fiji) and rotated to position the spindle poles horizontally. Subsequently, chromosome angles were determined by fitting a line along each chromosome axis and measuring the smallest angle between the chromosome axis and spindle pole-to-pole axis.

For EBP-2 imaging, a single z-section was acquired every 800 ms using 100 ms exposure, starting 1 min after NEBD. Kymographs were generated from a 5-pixel width line drawn pole-to-pole using KymographClear [54]. Summed-intensity projection (SIP) images were generated by adding 38 EBP-2 frames starting 1 min after NEBD and subtracting the background signal. Intensity profiles were generated from these SIPs, by drawing a rectangular box (1.5 x 22 µm) encapsulating both spindle poles and averaging of the pixel intensities of each column within.

bioRxiv preprint doi: https://doi.org/10.1101/2023.03.23.534015; this version posted March 24, 2023. The copyright holder for this preprint (which was not certified by peer review) is the author/funder, who has granted bioRxiv a license to display the preprint in perpetuity. It is made available under aCC-BY 4.0 International license.

Table S1: *C. elegans* Strains

STRAIN DESCRIPTION	SOURCE	IDENTIFIER
C. elegans N2 Bristol	Caenorhabditis Genetics Center	http://www.cgc.cbs.umn.edu/strain.php?id=10570
unc-119(ed3) III; ruIs32[pAZ132; pie-1/GFP::histone H2B] III; ddIs6 [GFP::tbg-1; unc-119(+)] V	Oegema et al. 2001, PMID: 11402065	TH32
unc-119(ed3) III; ltIs37 [pAA64; pie-1/mCherry::his-58; unc-119 (+)] IV		OD56
unc-119(ed3) III; ltSi120[[pDC170;Pndc-80:ndc-80 reencoded; cb-unc-119(+)]II #3	Cheerambathur et al.,2013, PMID: 24231804	OD611
unc-119(ed3) III; ltSi120[[pDC170;Pndc-80:ndc-80 reencoded; cb-unc-119(+)]II #3; ruIs32[pAZ132; pie-1/GFP::histone H2B] III; ddIs6 [GFP::tbg-1;unc-119(+)] V	Cheerambathur et al.,2013, PMID: 24231804	OD613
unc-119(ed3)III; ltSi129[pDC181;Pndc-80:NDC-80 (100,144,155AAA) reencoded; cb-unc-119(+)]II #2 ; ruIs32[pAZ132; pie-1/GFP::histone H2B] III; ddIs6 [GFP::tbg-1; unc-119(+)] V	Cheerambathur et al.,2013, PMID: 24231804	OD644
unc-119(ed3)III; ltSi122[pDC178;Pndc-80:NDC-80 (8,18,44,51AAAA) reencoded; cb-unc-119(+)]II #1; ruIs32[pAZ132; pie-1/GFP::histone H2B] III; ddIs6 [GFP::tbg-1; unc-119(+)] V	Cheerambathur et al.,2013, PMID: 24231804	OD688
unc-119(ed3)III; ltSi560 [pPLG014; Pmex-5::GFP::his-11::tbb-2_3'UTR, tbg-1::gfp::tbb-2_3'UTR; cb-unc-119(+)]V	Kim et al., 2016,PMID: 26953348	OD1702
unc-119(ed3)III; ltSi710[pDC267;Pndc-80:NDC-80 (66,96,100,125,144,155AAAAAA) reencoded; cb-unc-119(+)]II#1 ; ruIs32[pAZ132; pie-1/GFP::histone H2B] III; ddIs6 [GFP::tbg-1; unc-119(+)] V	Cheerambathur et al.,2017, PMID: 28535376	OD2312
dhc-1(lt45; dhc-1::gfp) I; unc-119(ed3) III?; ltIs37 [pAA64; pie-1/mCherry::his-58; unc-119 (+)] IV	This Study	OD2956
lt53[knl-1::GFP::tev::loxP::3xFlag)III; ltIs37 [pAA64; pie-1/mCHERRY::his-58; unc-119 (+)] IV	This Study	OD3075
lt53[knl-1::GFP::tev::loxP::3xFlag)III; ltSi711[pDC267;Pndc-80:NDC-80(66,96,100,125,144,155AAAAAA) reencoded; cb-unc-119(+)]II#1 ; ltIs37 [pAA64; pie-1/mCherry::his-58; unc-119 (+)] IV	This Study	OD3083
lt53[knl-1::GFP::tev::loxP::3xFlag)III; ltSi129[pDC181;Pndc-80:NDC-80 (100,144,155AAA) reencoded; cb-unc-119(+)]II #2; ltIs37 [pAA64; pie-1/mCherry::his-58; unc-119 (+)] IV	This Study	OD3121
klp-19(lt118[gfp::klp-19])III; ltIs3 [pAA64;pie1/mCherry::his-58; unc-119 (+)] IV	This Study	OD3192
ndc-80(lt54[ndc-80::GFP::tev::loxP::3xFlag])IV; unc-119(ed3) III?; ltIs37 [pAA64; pie-1/mCherry::his-58; unc-119 (+)] IV	This Study	OD3300
dhc-1(lt45; dhc-1::gfp) I; ltSi120[[pDC170;Pndc-80:ndc-80 reencoded; cb-unc-119(+)]II #3; unc-119(ed3) III?; ltIs37 [pAA64; pie-1/mCherry::his-58; unc-119 (+)] IV	This Study	OD3630
dhc-1(lt45; dhc-1::gfp) I; ltSi711[pDC267;Pndc-80:NDC-80 (66,96,100,125,144,155AAAAAA) reencoded; cb-unc-119 (+)]II#1; unc-119(ed3) III?; ltIs37 [pAA64; pie-1/mCherry::his-58; unc-119 (+)] IV	This Study	OD3631
knl-1(lt53[knl-1::GFP::tev::loxP::3xFlag]))III ltSi711 [pDC267;Pndc-80:NDC-80 (66,96,100,125,144,155AAAAAA) reencoded; cb-unc-119(+)]II#1; ltIs37 [pAA64; pie-1/mCHERRY::his-58; unc-119 (+)] IV	This Study	OD3633

bioRxiv preprint doi: https://doi.org/10.1101/2023.03.23.534015; this version posted March 24, 2023. The copyright holder for this preprint (which was not certified by peer review) is the author/funder, who has granted bioRxiv a license to display the preprint in perpetuity. It is made available under aCC-BY 4.0 International license.

dha6[ebp-2::mNG::::tev::loxP::3xFlag]II	This Study	DKC27
[ltSi597[pDC202 ; Pknl-1::mCherry::knl-1::knl-1 3'UTR; cb-unc-119(+)]; dhc-1 (lt45[dhc-1::gfp])]I; ltSi711[pDC267;Pndc-80:NDC-80(66,96,100,125,144,155AAAAAA) reencoded; cb-unc-119(+)]II#1; unc-119(ed3)III? ; ltIs37 [(pAA64) pie-1p::mCherry::his-58 + unc-119(+)] IV	This Study	DKC133
[dhaSi32[pDC646; Pknl-1::knl-1 reencoded (RRASA) :: mCherry::knl-13'UTR; cb-unc-119(+)]#1; dhc-1 (lt45[dhc-1 ::gfp])]I; ltSi711[pDC267;Pndc-80:NDC-80 (66,96,100,125,144,155AAAAAA reencoded; cb-unc- 119(+)]II#1; unc-119(ed3)III? ; ltIs37 [(pAA64) pie-1p ::mCherry::his-58 + unc-119(+)] IV	This Study	DKC157
ltIs37 [(pAA64) pie-1p::mCherry::his-58 + unc-119(+)] IV; (lt39[gfp::tev::loxP::3xFlag::mdf-1])V)	This Study	DKC231
ltSi120[[pDC170;Pndc-80:NDC-80 reencoded; cb-unc-119(+)]II #3; unc-119(ed3)III? ; ltIs37 [(pAA64) pie-1p::mCherry::his-58 + unc-119(+)] IV; (lt39[gfp::tev::loxP::3xFlag::mdf-1])V)	This Study	DKC232
ltSi711[pDC267;Pndc-80:NDC-80 (66,96,100,125,144,155AAAAAA) reencoded; cb-unc-119(+)]II#1; unc-119(ed3)III? ; ltIs37 [(pAA64) pie-1p ::mCherry::his-58 + unc-119(+)] IV; (lt39 [gfp::tev ::loxP :: 3xFlag::mdf-1])V)	This Study	DKC233
dha6[ebp-2::mNG::::tev::loxP::3xFlag]II; knl-3 (dha19 [mscarlet-I^3XFLAG::knl-3] V	This Study	DKC338
ruIs32 [pie-1p::GFP::H2B + unc-119(+)] III. ddIs6 [tbg-1::GFP + unc-119(+)] V.	This Study	DKC393
ruIs32 [pie-1p::GFP::H2B + unc-119(+)] III. ddIs6 [tbg-1::GFP + unc-119(+); he279[Δebp-1, ΔY59A8B.25, Δebp-3] V;	This Study	DKC394
spdl-1(dha113 (gfp::spdl-1))II knl-1(lt75[knl-1::mCherry]) III	This Study	DKC594
rod-1(dha112 (ROD-1::mScarlet-I))I; knl-1(lt53[knl-1 ::GFP ::tev::loxP::3xFlag]))III	This Study	DKC597
rod-1(dha112 (ROD-1::mScarlet-I))I; ruIs32 [pie-1p:: GFP::H2B + unc-119(+)] III. ddIs6 [tbg-1::GFP + unc-119(+)] V.	This Study	DKC760
spdl-1(dha113 (gfp::spdl-1))II; unc-119(ed3) III; ltIs37 [pAA64; pie-1/mCherry::his-58; unc-119 (+)] IV	This Study	DKC761

bioRxiv preprint doi: https://doi.org/10.1101/2023.03.23.534015; this version posted March 24, 2023. The copyright holder for this preprint (which was not certified by peer review) is the author/funder, who has granted bioRxiv a license to display the preprint in perpetuity. It is made available under aCC-BY 4.0 International license.

Table S2: CRISPR gRNAs

Gene No.	Name	Allele Generated	guideRNA sequence	References
Y43F4B.6	*klp-19*	*klp-19 (it118[gfp::klp-19])III*	ACCATTCATAGGCCGAGCA	Direct Integration (Waaijers et al., 2013)
C02F5.1	*knl-1*	*knl-1(lt53[knl-1::gfp::tev::loxP::3xFlag])III*	TCGAATGCTGGTGTTCTCTA	SEC (Dickinson et al., 2015)
T21E12.4	*dhc-1*	*dhc-1 (lt45[dhc-1::gfp])I*	CTACCAACGAGGAGTTGCAT	Direct Integration (Waaijers et al., 2013)
VW02B12L.3	*ebp-2*	*ebp-2 (dha6[ebp-2::mNG::tev::loxP::3xFlag])II*	GCAGGCAAATCTGGACGATA	SEC (Dickinson et al., 2015)
C06A8.5	*spdl-1*	*spdl-1(dha113 [gfp::spdl-1])II*	aatcagtATGCCTGACGACG	Ribonucleoprotein complex (Paix, et al., 2015)
F55G1.4	*rod-1*	*rod-1(dha112 [rod-1::mSc-I])I*	caacgaatttatTTAAGAGT	Ribonucleoprotein complex (Paix, et al., 2015)

Table S3: dsRNAs used in this study

Gene No.	Name	Oligonucleotide (5'-3'), #1	Oligonucleotide (5'-3') #2	Template
Y43F4B.6	*klp-19*	5'- aattaaccctcactaaagg ATTGGGAGAGCTGGTGAATG-3'	5'- taatacgactcactatagg GACTTTCCTACGTGCTTCGC-3'	N2 genomic DNA
C02F5.1	*knl-1*	5'- aattaaccctcactaaagg TTCACAAACTTGGAAGCCGCTG -3'	5'- taatacgactcactatagg AATCTCGAATCACCGAAATGTC -3'	N2 genomic DNA
W01B6.9	*ndc-80*	5'- aattaaccctcactaaagg GATGACAAGTACATTCAGAGATTATACAAATGATC-3'	5'- taatacgactcactatagg GTGGTTCAAGATTCATTTGAATATTAAGTCCACTG-3'	N2 genomic DNA
F55G1.4	*rod-1*	5'- aattaaccctcactaaagg AATGCAAATCTTTTTGGATGGGAGAAAC-3'	5'- taatacgactcactatagg CATCGACGAATTTGATTCGATCAATC-3'	N2 genomic DNA
Y69A2AR.30	*mdf-2*	5'- aattaaccctcactaaagg GTGAACTGACGTCGAGAATGAG-3' -3'	5'- taatacgactcactatagg GACGGATGTAAAGACACAAAACG-3'	N2 genomic DNA
		Lowercase letters denote T3 and T7 sequences included for RNA synthesis.	Lowercase letters denote T3 and T7 sequences included for RNA synthesis.	
